# Pressure effect on impurity local vibrational mode and phase transitions in *n*-type iron-doped indium phosphide

**DOI:** 10.1038/s41598-018-19679-2

**Published:** 2018-01-19

**Authors:** Chih-Ming Lin, I-Jui Hsu, Sin-Cheng Lin, Yu-Chun Chuang, Wei-Ting Chen, Yen-Fa Liao, Jenh-Yih Juang

**Affiliations:** 1 Department of Physics, National Tsing Hua University, Hsinchu, 30013 Taiwan; 2 Department of Molecular Science and Engineering, National Taipei University of Technology, Taipei, 10608 Taiwan; 3 National Synchrotron Radiation Research Center, Hsinchu, 30076 Taiwan; 4 Department of Electrophysics, National Chiao Tung University, Hsinchu, 30050 Taiwan

## Abstract

The evolution of iron local vibrational mode (Fe LVM) and phase transitions in *n*-type iron-doped indium phosphide (InP:Fe) were investigated at ambient temperature. *In-situ* angle-dispersive X-ray diffraction measurements revealed that InP:Fe starts to transform from *zinc-blende* (ZB) to *rock-salt* (RS) structure around 8.2(2) GPa and completes around 16.0(2) GPa. The Raman shift of both transverse and longitudinal optical modes increases monotonically with increasing pressure, while their intensities become indiscernible at 11.6(2) GPa, suggesting that the pressure-induced phase transition is accompanied by significant metallization. In contrast, originally absent at ambient pressure, the Raman shift of Fe LVM appears at ∼420 cm^−1^ near 1.2 GPa and exhibits a dome shape behavior with increasing pressure, reaching a maximum value of ∼440 cm^−1^ around 5 GPa, with an apparent kink occurring around the ZB-RS transition pressure of ∼8.5(2) GPa. The Fe K-edge X-ray absorption near edge structure (XANES) confirmed the tetrahedral site occupation of Fe^3+^ with a crystal field splitting parameter Δ_*t*_ = 38 kJ·mole^−1^. Our calculations indicate that the energy parameters governing the phase transition are Δ_*t* = _0.49 and Δ_*o*_ = 1.10 kJ·mole^−1^, respectively, both are much smaller than Δ_*t*_ = 38 kJ·mole^−1^ at ambient.

## Introduction

Indium phosphide (InP) has been a prominent compound semiconductor in arrays of advanced information technology applications, such as high-electron-mobility transistors (HEMTs), impact avalanche transit-time (IMPATT) diodes, transferred electron devices (TEDs), optical amplifiers, lasers, etc. In particular, InP is widely used as the substrate material for both InP-based devices and optoelectronic devices based on strained pseudomorphic heterojunction structures. For example, very recently, Chang *et al*.^1^ demonstrated that a 2 nm-thick pure InAs channel grown on InP substrate could give rise to an unprecedentedly high value of current-gain cutoff frequency (*f*_T_) (up to 710 GHz). Subsequently, semi-insulating (SI) InP substrates are widely used for fabricating high-frequency optoelectronic devices and integrated circuits. Among various SI InP substrates, the iron-doped InP (InP:Fe) single crystal has received extensive attention due to its superior thermal stability^2^. Iron doped InP is a technologically relevant material and has grown by various methods, like as high-temperature ion implantation^3^ and liquid encapsulated Czochralski (LEC)^4^. In InP:Fe, the iron atom occupies the indium site substitutionally and primarily adopts the neutral $F{e}_{In}^{3+}$ state (labelled 3+ to denote its oxidation state). However, in the presence of shallow donors some of the irons might be compensated, leading to the existence of more negatively charged state, i.e. Fe^2+^, and hence altering the local symmetry of doped iron atoms^2^. Thus, one of the essential issues is to identify the exact oxidation states of the doped iron atoms and clarify its influence on structural transitions under external pressure or strains. In this respect, we have carried out Fe K-edge X-ray absorption near structure (XANES) and extended x-ray absorption fine structure (EXAFS) analyses to confirm the electronic structure and local geometric symmetry of the doped Fe atoms. Moreover, upon the incorporation of doped atoms, the lattice parameters of the parent phase can be either contracted or expanded. In the present case, since the ionic radius of Fe^3+^ (0.49 Å) is smaller than that of In^3+^ (0.62 Å)^5^, thus, one expects that Fe-doping will result in contraction (or shortening) of the InP unit cell parameters. This may, in turn, affect the structure of the epitaxial layers grown on InP:Fe substrates. Consequently, it is of essential importance to comprehend how the strain (or stress) introduced by doping affects the intrinsic structural, electrical and optical properties of InP:Fe.

The electronic, structural and vibrational properties of semiconductors are significantly altered by the introduction of impurities. Larkin^6^ and McCluskey^7^ reported that the translational symmetry might be broken and one or more new vibrational modes might appear when impurity was introduced. For example, if an impurity with smaller mass replaces a heavier host atom, its vibrational frequency will lie above the phonon frequency range of the original mode. Nevertheless, unlike a phonon, the defect-induced vibrational mode is localized in real space and frequency domain, and is usually referred to as a *local vibrational mode* (LVM)^6,7^. By the same token, it is anticipated that upon doping when Fe atoms replace the In atoms on the lattice site the translational symmetry of the InP crystal may be interrupted, giving rise to a new localized vibration mode, which will be labeled as Fe LVM in the present study. Thus, by searching for such Fe LVM and its dependence on the applied pressure, one might be able to gain more information on the pressure-induced structural changes, as well.

As one of the primary thermodynamic parameters, pressure can “modify” the physical properties of a material, such as inducing structural, optical, electronic and magnetic transitions, in remarkable fashions. The pioneering work that reported the pressure-induced structural phase transitions in InP was reported by Minomura *et al*.^8^ which indicated a transition to metallic phase at around 13 GPa. Subsequently, Jamieson^9^ further pointed out that the transition was from zinc-blende (ZB) structure (space group $F\bar{4}3m$) to *rock-salt* (RS) structure (space group $Fm\bar{3}m$) accompanied by a volume change of 19.6%. By using angle-dispersed X-ray-diffraction (ADXRD) method, Menoni *et al*.^10^ and McMahon *et al*.^11^ reported that the transition from ZB to RS structure took place at 10.80(5) and 9.8(5) GPa, respectively. Based on optical absorption spectroscopy, Müller *et al*.^12^ and Whitaker *et al*.^13^ indicated that the transition occurs at 10.15(5) and around 9.9(1) GPa, respectively. On the other hand, Kobayashi *et al*.^14^ and Ernst *et al*.^15^ deduced a transition occurring at 10.35(5) and 11.2(4) GPa, respectively, from photoluminescence measurements and Whitaker *et al*.^13^ indicated a transition occurring around 9.9(1) GPa from Raman measurements. As mentioned above, an average reference point of 10.67(5) GPa is taken as the ZB-to-RS phase transition pressure of bulk InP.

Recently, x-ray absorption (XANES and EXAFS) and proton induced x-ray emission (PIXE) studies were performed on InP:Fe by Cesca *et al*.^3^. The results indicated that the high-temperature implantation process favored the incorporation of iron atoms in high-symmetry octahedral sites. Nonetheless, the subsequent high-temperature annealing controls the kick-out of the iron atoms from substitutional locations, leading to ejection of point defect flux and formation of Fe-P complexes. The displacement of the iron atoms were further found to favor the formation of Fe-P complexes containing few atoms with an increased coordination (octahedral) with respect to the tetrahedral coordination exhibited in the as-implanted samples.

In this work, by combining the results obtained from ADXRD and Raman measurements, the pressure-induced phase transition in bulk InP:Fe grown by the liquid encapsulated Czochralski (LEC) method over the pressure range of 8.2(2)–14.6(2) GPa is systematically investigated. The consistencies of the results simultaneously obtained from the two independent methods should shed some lights on resolving the underlying reason resulting in the scattered results reported previously.

## Results and Discussion

**Figure 1:**
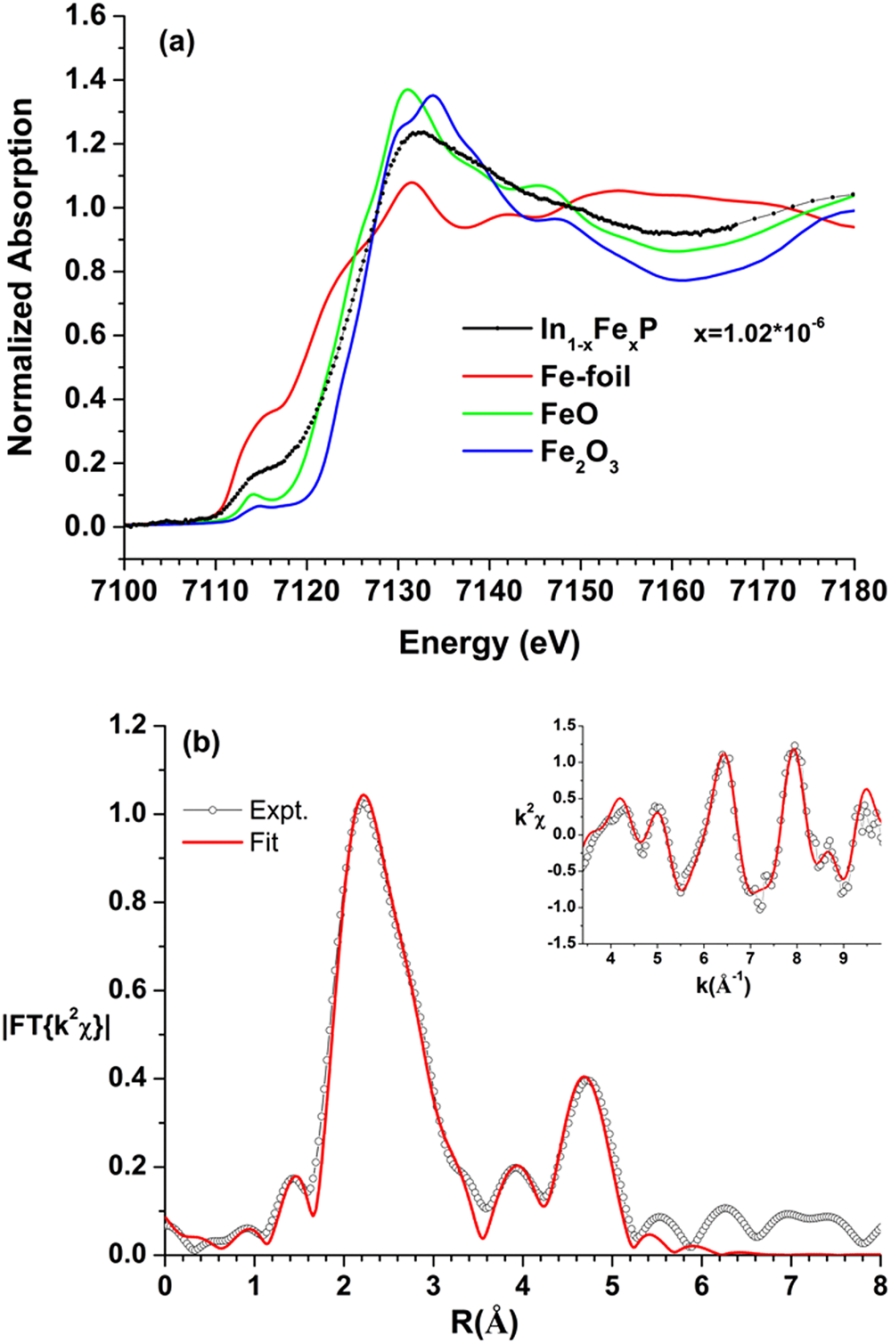
(**a**) Fe K-edge XANES of In_1-x_Fe_x_P (x = 1.02 × 10^−6^) and some reference samples. (**b**) EXAFS fitting results (*k*^2^χ shown in inset). Open circles and solid lines denote experimental data and fitting results, respectively. The insets show the *k*^2^-weighted EXAFS raw data (*k*^2^χ) used in the analysis.

As mentioned above that, when the doped Fe atom replaces the In atom, its exact valence state may lead to very different local symmetries at the original site of In atom. Thus, it is essential to ensure the actual valence state of the doped Fe atoms by carrying out the XANES measurements. The Fe K-edge XANES of In_1-x_Fe_x_P (x = 1.02 × 10^−6^) together with that of pure Fe-foil, FeO, and Fe_2_O_3_ are displayed in Fig. 1(a) and the corresponding first derivative spectrum is depicted in Figure S1. Based on the features of rising edge region and the first derivative spectra, it is suggested that the oxidation state of Fe in In_1-x_Fe_x_P (x = 1.02 × 10^−6^) is closer to that of Fe_2_O_3_ and hence the valence state is likely to be Fe^+3^(3*d*^5^). Moreover, it is noted that a pre-edge peak around 7114.1 eV associated with the 1 s (Fe)→3*d* (Fe) transition can be observed in the In_1-x_Fe_x_P sample. In general, this is a forbidden transition according to the dipole selection rule. Thus, the appearance of this pre-edge peak indicates that the Fe doping may have led the FeP_4_ T_d_ local geometry to become non-central symmetrical, which strongly suggests that the doped Fe atoms are primarily taking on the in site of the ZB-structured InP as previous works of the atomic environment of iron impurities introduced in InP by high-temperature ion implantation^3^. Cesca *et al*.^3^ indicated that the Fe atoms in tetrahedral interstitial sites were treated as if they contributed to the random fraction in the whole range of angular coordinates.

Taking a closer look at the pre-edge features and comparing to the previous report by Ney *et al*.^16^ we noticed that some Fe-Fe related Fe clusters might have contributed to the XANES spectrum. In order to gain more quantitative information on the effects of Fe-doping, the EXAFS measurement was carried out and the results of data analysis were displayed in Fig. 1(b) and in Table 1. The results indicate that the spectrum is fit best by a combination of ~27(3)% of Fe cluster with ~73(3)% of In_1-x_Fe_x_P. Based on the obtained Fe-Fe distances and the corresponding coordination numbers in fitting of the first shell, the Fe clusters are mainly packed with the body center cubic (BCC) structure. The obtained bond length for the four coordination number Fe-P is about 2.28(1) Å, which is close to the Fe-P bond lengths in FeP or FeP_2_ with distances between 2.243 and 2.350 Å^17,18^. Meanwhile, our results is also comparable to the previous reported by Cesca *et al*.^3^, which the averaged Fe-P distance of tetrahedral geometry is around 2.36(2) Å. Moreover, this tetrahedral geometry of local Fe site also consistent with the XANES line shape analysis in pre-edge peak around ~7114 eV. The interatomic distance ~4.55(6) Å between Fe and In atoms provides a further evidence of Fe doped into InP structure. Based on the XANES and EXAFS results, we believe that the oxidation state of Fe in InP structure is mainly in Fe^3+^ and the Fe is bonded with four P atoms in tetrahedral geometry^19^. However, it is noted that, since the EXAFS spectra were taken with the fluorescence mode, the weighted EXAFS χ function fit based on the back scattering paths generated by known model in FEFF program adopted here is reflecting the possible contributions to the EXAFS signal from local short-range-ordering structures rather than indicating the volume concentration percentages of two different constituent phases. Otherwise, it would be readily observed in the ADXRD measurements to be discussed below.

**Table 1:** Summary of EXAFS fitting results: coordination numbers (C.N.), mean square relative displacements (σ^2^), and interatomic distance (R).

Phase	BCC-Fe			In_1-x_Fe_x_P		
ratio	0.27(3)			0.73(3)		
	R (Å)	σ^2^ (Å^2^)	C.N.	R (Å)	σ^2^ (Å^2^)	C.N.
Fe-P				2.28(1)	0.006(1)	4
Fe-Fe	2.40(1)	0.006(1)	8			
Fe-Fe	2.92(3)	0.008(3)	6			
Fe-Fe	4.02(5)	0.006(2)	4			
Fe-In				4.55(6)	0.006(2)	4
Fe-Fe				4.76(6)	0.006(2)	4
k- range (Å^−1^)	[3.4, 9.8]					
r- range (Å)	[1.78, 4.97]					
*R*_fit_ factor	1.1%					

**Figure 2:**
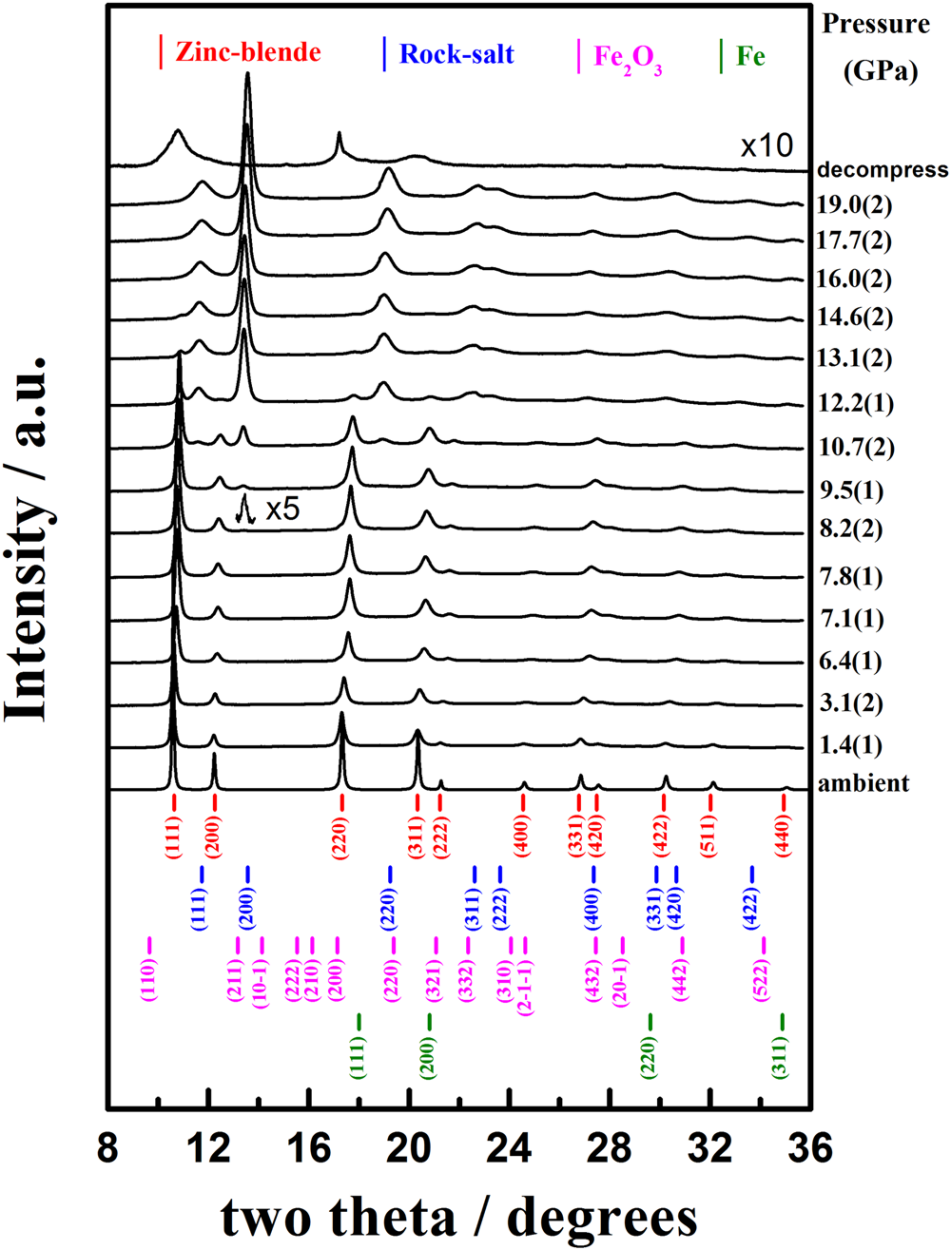
Representative ADXRD patterns of bulk InP:Fe at elevated pressures. Bulk InP:Fe exhibited a phase transition with an onset pressure of 8.2(2) GPa.

Figure 2 shows the representative pressure-dependent room-temperature *in situ* ADXRD spectra of bulk InP:Fe with applied pressures up to 19.0(2) GPa. It is evident from Fig. 2 that single phase ZB structure persists up to a pressure of 7.8(1) GPa and a mixture of $F\bar{4}3m$ and $Fm\bar{3}m$ space groups is observed with pressure up to 14.6(2) GPa. Furthermore, as is evident from Fig. 2, the ADXRD patterns at all pressures show no trace of iron and/or ferric oxide clusters formed in the present InP:Fe, suggesting a doping of Fe atoms within the InP lattice. The *d*-spacings of the eleven reflections, namely (111), (200), (220), (311), (222), (400), (331), (420), (422), (511), and (440), of the $F\bar{4}3m$ space group under ambient pressure are 3.3468, 2.8979, 2.0503, 1.7489, 1.6744, 1.4500, 1.3312 1.2966, 1.1846, 1.1174, and 1.0267 Å, respectively. The unit-cell parameters and the interatomic bond length (bonding distance) in In(Fe)-P at ambient pressure determined from Fig. 2 are *a* = 5.8292(1) Å, V/Z = 49.518(3) Å^3^ (Z = 4) and 2.524 Å, respectively. These values are smaller than that of parent pure InP, where a = 5.8682(1) Å, V/Z = 50.518(3) Å^3^ and In-P bond length ≈ 2.541(1) Å were obtained, respectively (Figure S2). Moreover, they are also smaller than the values of a = 5.8687(5) Å and V/Z = 50.532 Å^3^ listed in ICSD No. 600858, as well as In-P bond length ≈ 2.541 Å in pure InP reported by Martin^20^. The corresponding contraction in the lattice constant and bond length is ~0.6%, which is much higher than the expansion (~0.02%) measured, for example, for S doped InP^21^. It is quite surprising to observe such a large lattice contraction induced by such a small dopant concentration (x = 1.02 × 10^–6^). Nevertheless, since the crystal ionic radius for Fe^3+^ (0.49 Å) is much smaller than that of In^3+^ (0.62 Å)^7^, it is plausible to expect that substantial contraction of the lattice parameter may occur when iron atoms substitute for indium atoms at the tetrahedral sites and lead to a negative strain component. In fact, Cesca *et al*.^3^ in their high-temperature implantation of Fe in InP had found that the doped iron could present in a number of local configurations, which are not ordered beyond the first coordination shell. In their as-implanted samples, a dominant coordination number (CN) close to 4 was observed with a Fe-P bond length of about 2.243 Å. Comparing with the lattice constant and bond distance of BCC structured Fe, which are 2.856 Å and 2.483 Å, respectively^22^, it is suggestive that the negative strain component induced by Fe-doping can be indeed very significant. Moreover, Bachmann *et al*.^23^ and Iseler^24^ reported that there existed substantial amount of dislocations and twins in InP crystals prepared by liquid encapsulated Czochralski methods, which might also have some effects in facilitating clustering of the doped Fe atoms. As mentioned above, our XANES/EXAFS results indicated the possible existence of tiny Fe clusters in the present InP:Fe crystal. Together with the fact that no impurity phases were discernible (see Fig. 2), it is suggestive that the doped Fe atoms are primarily residing at the tetrahedral sites (CN∼4) to replace In atoms and form tiny Fe-P complexes (*d*(In-P) = 2.538 Å reduced to *d*(Fe-P) = 2.28(1) Å) similar to that observed in high-temperature Fe-implanted InP by Cesca *et al*.^3^, and form tiny Fe clusters, which, in turn, would result in substantial negative strain component and account for the observed large contraction of the lattice parameter (~0.6%).

The calculated theoretical density of 4.887(1) g · cm^−3^ is slightly larger than the value of 4.789(1) g · cm^−3^ listed in the ICSD No. 600858. Although one might argue that the absence of any iron or Fe-associated complex clusters directly from the ADXRD spectra displayed in Fig. 2 might just be due to the fact that the doping concentration of Fe is relatively low, nevertheless, the noticeable changes in the ZB structure evidently displayed above strongly indicated that in the present case the doping is achieved.

As the pressure reaches 8.2(2) GPa, a discernible new diffraction peak corresponding to the (200)-reflection of $Fm\bar{3}m$ starts to emerge at the higher two-theta side of the reflection (200) of the $F\bar{4}3m$ for bulk InP:Fe. Miao *et al*.^25^ reported that, for InP, the transition from ZB to RS at high-pressure often involves an intermediate transition state, which appeared to be rather universal in the sense that its position along the path and the corresponding geometry was independent of the chemical components of the semiconductor. As the pressure is further increased to 9.5(1) GPa, the reflections (111), (220), (311), (222) and (400) of the $Fm\bar{3}m$ appeared at the higher two-theta side of the reflections (111), (220), (311), (222) and (331) of the $F\bar{4}3m$ space group, respectively. When pressure up to 10.7(2) GPa, the reflections (331), (420) and (422) of the $Fm\bar{3}m$ appeared at the higher two-theta side of the reflections (422) and (511) of the $F\bar{4}3m$ space group. Moreover, although the intensity of the new peak grows substantially with the increasing pressure, coexistence of diffraction peaks from the original $F\bar{4}3m$ is evident over a wide range of pressures (8.2(2) ∼ 14.6(2) GPa), indicating that the phase transition is taking place locally rather than globally. The phase transition is likely taking place via the nucleation and growth process. Namely, during the phase transition, new phase appears as localized nuclei at the onset pressure of ∼8.2(2) GPa and then grows with further increase in applied pressure, instead of taking place in a globally catastrophic fashion at certain onset pressure. Based on the thermodynamics arguments that the transition pressure should be close or equal to the onset pressure. Thus, in the present case, the transition pressure of ZB to RS structure for bulk InP:Fe is taken as 8.2(2) GPa. The onset transition pressure of 8.2(2) GPa apparently is much smaller the values of 9.8(5) GPa∼10.80(5) GPa reported for pristine InP^8–11^, not to mention the magnitude of 13 GPa reported by Minomura *et al*. in their pioneering measurement^6^. The much reduced onset transition pressure is believed to result from the lattice disorders and accompanying internal negative strain induced by Fe-doping^3^. (See below) The reflections of the ZB structure disappear completely leaving only the reflections of the RS structure appearing in the ADXRD spectra for pressure beyond 16.0(2) GPa, indicating that the phase transition occurring over the whole sample volume. The lattice parameters at 8.2(2) GPa for the present bulk InP:Fe are *a* = 5.7153(2) Å and V/Z = 46.672(6) Å^3^ (Z = 4) for the ZB structure and *a* = 5.3047(6) Å, V/Z = 37.318(8) Å^3^ (Z = 4) for the RS structure, respectively, implying that the transition from ZB to RS structure is accompanied by a substantial volume decrease of ~15.7%. The experimental total enthalpy change (Δ°*H*) for the ZB to RS structure transformation at 8.2(2) GPa may be estimated as follows^26^: Δ°*H* ≈ *P*_tr_ (−Δ*V*) = 8.2 GPa × 9.354 Å^3^ × 6.02 × 10^23^/mole = 46.18 KJ·mole^−1^, which can offer enough amount of internal energy for reconstructing the RS structure. The calculated theoretical densities are 5.186(1) and 6.485(1) g · cm^−3^ for ZB and RS structures at 8.2(2) GPa, respectively, and both are increased with increasing applied pressure. It is noted that the theoretical density of RS structure is higher than that of the ZB structure under high pressure. When the pressure is relaxed back to the ambient pressure (decompress), a tremendous amount of strain is expected to be released in the sample and a substantial part of the InP:Fe reverted to the ZB structure. Nevertheless, as is evident from the decompressed ADXRD pattern shown in Fig. 2, there is still show no trace of iron and/or ferric oxide clusters formed in the present InP:Fe. Even more interestingly, the corresponding diffraction peaks of the ZB structure appear to noticeable broadening. This is indicative that the pressure-induced phase transition path in the present InP:Fe could be highly reversible.

One possible reason for the noticeable reduction in the onset pressure of phase transition may be due to the degraded crystal ordering induced by Fe doping, which reduces the stability of the ZB crystal structure. To explain the reduction of the phase-transition pressure of bulk InP:Fe, it is heuristic to compare the behaviors observed in diluted magnetic semiconductors (DMS’s) with similar crystal structures. Maheswaranathan *et al*.^27^ reported that, in cadmium telluride (CdTe), substituting zinc with cadmium in the ZB lattice evidently led to more stable lattice in Cd_0.52_Zn_0.48_Te than with manganese in Cd_1-*x*_Mn_*x*_Te with 0 ≤ *x* ≤ 0.52. They also disclosed that manganese was responsible for making the ZB crystal structure more susceptible to the applied pressure instead of zinc. In that the relatively loosely bounded 3*d* electrons in manganese are preferably hybridized into the tetragonal bonds. On the contrary, the cadmium and zinc *d* levels do not hybridize with the *sp*^3^ bonding orbitals. Consequently, it is plausible to expect that the reduction of the phase transition pressure observed in the present iron-doped InP may be caused by the similar hybridization between the iron 3*d* orbitals and the original tetrahedral bonds. This 3*d* orbital hybridization tends to force the transition of ZB to RS structure and hence reduces the onset pressure of the phase transition. Alternatively, the significant difference between the ionic size of Fe^3+^ and In^3+^ is also expected to result in tremendous local lattice strain, which may in turn reduces the stability of ZB phase of bulk InP:Fe under the applied external pressure. It is, nevertheless, quite surprising that such a drastic change can be realized even with an iron concentration of ∼10^16^ cm^−3^, which corresponds to replacing only 1 in ∼ 2 × 10^6^ indium atoms with an iron atom.

**Figure 3:**
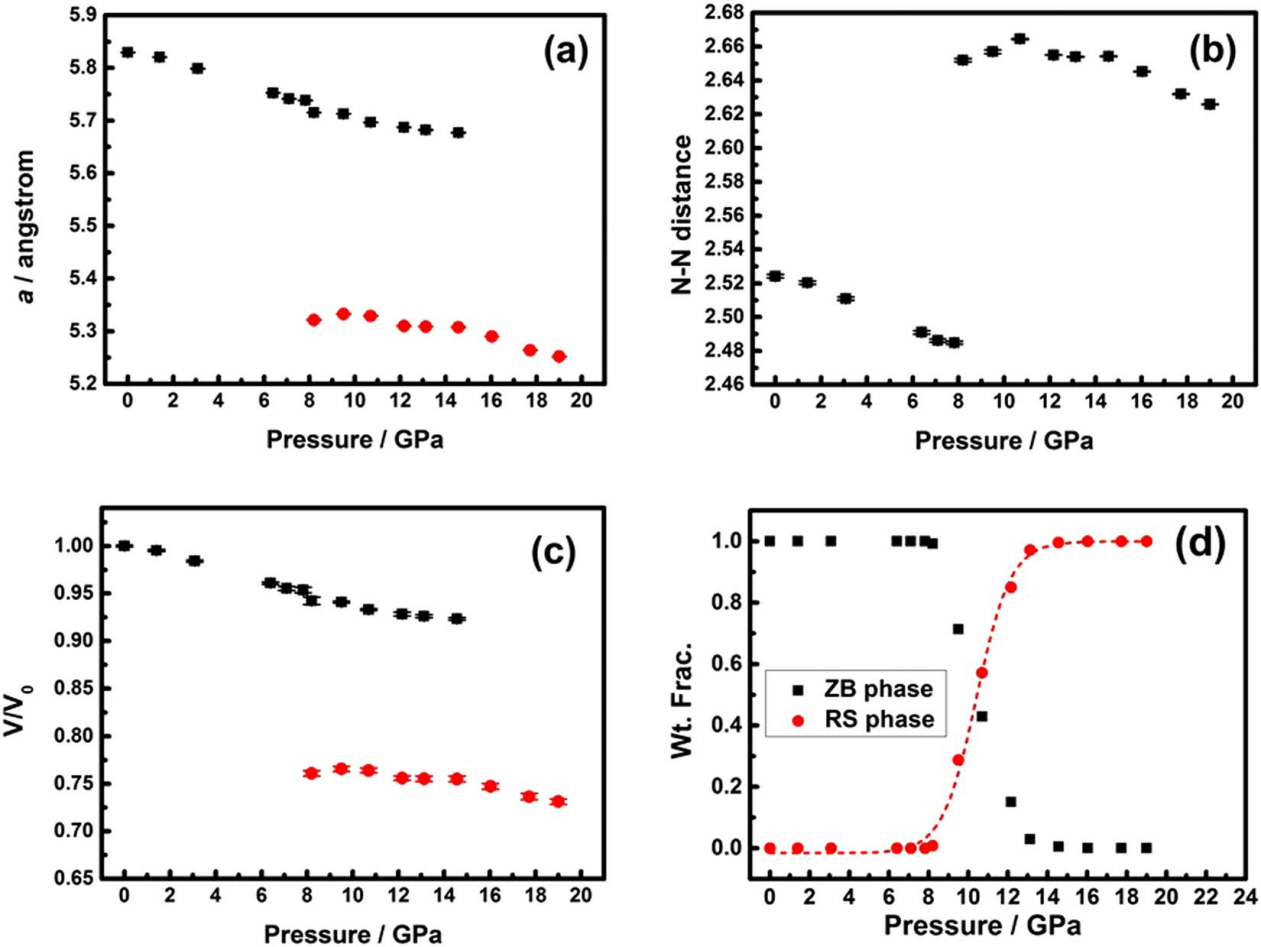
(**a**) Pressure dependence of the *a* of bulk InP:Fe at 300 K. (**b**) Pressure dependence of the *a* of bulk InP:Fe at 300 K. (**c**) Pressure dependence of the V/V_0_ of bulk InP:Fe at 300 K. (**d**) The weight fraction (Wt. Frac.) curves of $F\bar{4}3m$ and *Fm*3*m* space groups versus pressure at 300 K.

Figure 3(a) shows the variation of lattice parameters for the ZB $(F\bar{4}3m)$ and RS $(Fm\bar{3}m)$ phases as a function of the applied pressure, respectively. From the results, it can be derived that, at *P* = 8.2(2) GPa, the values of the linear compressibility along the ***a***-axis (*K*_*a*_ = −(1/*a*)(*da*/*dP*)_*T*_) are 2.144 × 10^−3^ GPa^−1^ and 8.643 × 10^–4^ GPa^−1^ for ZB and RS phases, respectively. Although it is quite surprising that the linear compressibility of the ZB phase is about 2.5 times higher than that of the RS phase, it is, nevertheless, qualitatively consistent with bond length dependent bulk modulus tendency predicted by Cohen^28^. The higher *K*_*a*_ exhibited in the ZB phase indicates that its lattice is more susceptible to compressive stress than that of the RS phase. Indeed, as shown in Fig. 3(b), the nearest neighbor (N-N) distance of the ZB phase displays significant pressure dependence. The N-N distance can also be denoted as the bonding distance in In(Fe)-P. Consequently, the results shown in Fig. 3(b) implies that the pressure dependent compressibility of the nearest neighbor distance is essentially linear, with *K*_*N-N*_ ≡ {−[1/(*N-N*)_0_][*d*(*N-N*)/*dP*]_*T*_} ≈ 2.139 × 10^−3^ GPa^−1^ up to 7.8(1) GPa. Here, (*N-N*)_0_ is the N-N distance at ambient pressure. Figure 3(b) also shows that the N-N distance in the RS phase is longer than that in the ZB phase. Similar results were also observed in CdS under high pressure^25^, where the bond lengths were 2.53 Å and 2.71 Å for tetrahedrally bonded ZB phase and octahedrally bonded RS phase, respectively. Figure 3(c) shows the volume versus pressure (*V*(*P*)) data measured at the ambient temperature for the present InP:Fe. Below 7.8(1) GPa, the *V*(*P*) of the ZB phase can be satisfactorily fitted by a quadratic polynomial expression: *V*(*P*) ≈ 198.34 − 1.017 *P* − 0.030*P*^2^. From the expression, the volume compressibility (*K*_*V*_ ≡ [−(1/*V*_0_)(*dV*/*dP*)_*T*_]) can be obtained to give *K*_*V*_ ≈ 6.352 × 10^−3^ GPa^−1^ up to 7.8(1) GPa. The fact that both the nearest neighbor distances and the cell volume of the ZB phase decrease linearly with the increasing hydrostatic pressure up to 7.8(1) GPa indicates that within this pressure range the lattice deforms elastically without introducing significant dislocation movements or twinning effect. Figure 3(d) shows the respective weight fraction (Wt. Frac.) curves of ZB and RS structures as a function of the applied pressure. The weight fraction of both the ZB and RS structures does not change noticeably until the pressure is reaching the intermediate pressure range of 8.2(2)–14.6(2) GPa. A closer examination indicates that the weight fraction of the RS phase remains essentially zero below 8.2(2) GPa and then increases slightly to ∼0.008 at 8.2(2) GPa. Thereafter, the weight fraction value increases quickly up to ∼1.00 at 16.0(2) GPa, beyond which the ZB phase has evidently completely converted to the RS phase. The existence of a pressure range of continuous phase change displayed in Fig. 3(d) indicates that the pressure-induced structural phase change between ZB and RS phases is taking place locally rather than globally. The pressure at which both phases have equal weight fraction is determined to be 10.4(1) GPa by a sigmoidal fit of Boltzmann function, as indicated by the dashed lines and the dashed arrow in Fig. 3(d).

The other distinct anomaly to be noted in Fig. 3(a)–(c) is that at a pressure of 8.2(2) GPa the ZB phase seems to undergo a discontinuous drop in the lattice constant, N-N distances, and V/V_0_, which has been reported previously in ZnSe_x_Te_1-x_ system under high pressure^29^. Although the underlying mechanism remains to be clarified and further investigations are certainly needed to clarify this issue. Nevertheless, we believe that one possible reason might be arising from the elastic-to-plastic deformation transition and the associated defect activities beyond some particular pressure. Alternatively, it might have been originated from the pressure-induced coordination transferring near the phase transition boundary. The interactions between the iron ion and the InP are purely electrostatic (ionic). In our case here, the Fe^3+^ occupies the tetrahedral site at the center of the cubic ZB structure. Due to the lack of a center of symmetry for the tetrahedron, the *d* orbitals of Fe^3+^ split into the two groups of orbitals, namely the lower doublet *e* and upper triplet *t*_2_, which are separated by an energy given by the tetrahedral crystal field splitting parameter Δ_*t*_^30^. The iron has *d*^6^*s*^2^ valence electron configuration and becomes Fe^3+^ in In_1-x_Fe_x_P. On the other hand, when the Fe^3+^ occupies the central symmetric octahedral site at the center of the cubic RS structure, the *d* orbitals of Fe^3+^ split into the upper doublet *e*_*g*_, the x^2^–y^2^ and z^2^ orbitals, and lower triplet *t*_*2g*_, the xy, az, and yz orbitals. In this case, the *t*_*2g*_ and *e*_*g*_ orbitals are separated by an energy determined by the octahedral crystal field splitting parameter, Δ_0_. The crystal field splitting in the tetrahedral field is intrinsically smaller than in the octahedral field. There are 4 and 6 ligands in the tetrahedral and octahedral complex, respectively, and hence the ligand field of the tetrahedral field is roughly $\frac{2}{3}$ of the octahedral field. The direction of ligand approach in tetrahedral complex does not coincide with the d-orbitals. This reduces the field by a factor of $\frac{2}{3}$. Therefore Δ_*t*_ is roughly $\frac{2}{3}\times \frac{2}{3}=\frac{4}{9}$ of Δ_0_. Burns^30^ suggested that, for Fe^3+^, the value of Δ_*o*_ should be larger than that of Δ_*t*_, and based on simple electrostatic arguments and by group theory it can be shown that ${{\rm{\Delta }}}_{o}=\frac{9}{4}{{\rm{\Delta }}}_{t}$ and ${{\rm{\Delta }}}_{o}-{{\rm{\Delta }}}_{t}=\frac{5}{4}{{\rm{\Delta }}}_{t}$. It is suggestive that the pressure-induced coordination-mismatch factor in the present *n*-type bulk InP:Fe SI may have triggered some structural accommodation activities, such as dislocation nucleation, leading to the discontinuous drop observed here.

At 8.2(2) GPa, an intermediate transition state, mixed distorted ZB and initial RS structures. Then, the Fe^3+^ exists in mixed distorted tetrahedral and initial octahedral fields. The extrapolated volume at 8.2(2) GPa by using the *V*(*P*) expression for ZB structure below 7.8(1) GPa described above is 187.972(1) Å^3^. Comparing to the volume of 186.687(3) Å^3^ obtained at 8.2(2) GPa of ZB structure experimentally, the difference of the total enthalpy change (Δ°*H*) for the ZB to RS structure transformation at 8.2(2) GPa may be estimated as follows^26^: Δ°*H* ≈ *P*_tr_(−Δ*V*) = 8.2 GPa × 0.321 Å^3^ × 6.02 × 10^23^/mole = 1.59 kJ·mole^−1^. This amount of energy should enough to create the distorted tetrahedral and initial octahedral fields. Then, 1.59 kJ·mole^−1^ = ${{\rm{\Delta }}}_{t}+{{\rm{\Delta }}}_{o}=\frac{13}{4}{{\rm{\Delta }}}_{t}=\frac{13}{9}{{\rm{\Delta }}}_{o}$. The energies of the tetrahedral and octahedral parameters for transforming from ZB to RS structure at 8.2(2) GPa can also be estimated to give Δ_*t*_ = 0.49 and Δ_*o*_ = 1.10 kJ·mole^−1^, respectively. This value is much smaller than that of Δ_*t*_ ~ 38 kJ·mole^−1^ at ambient, which indicates that the increasing of external pressure can significantly reduce the crystal field strength Δ_*t*_ of the ZB phase and facilitate the phase transformation to the RS phase.

**Figure 4:**
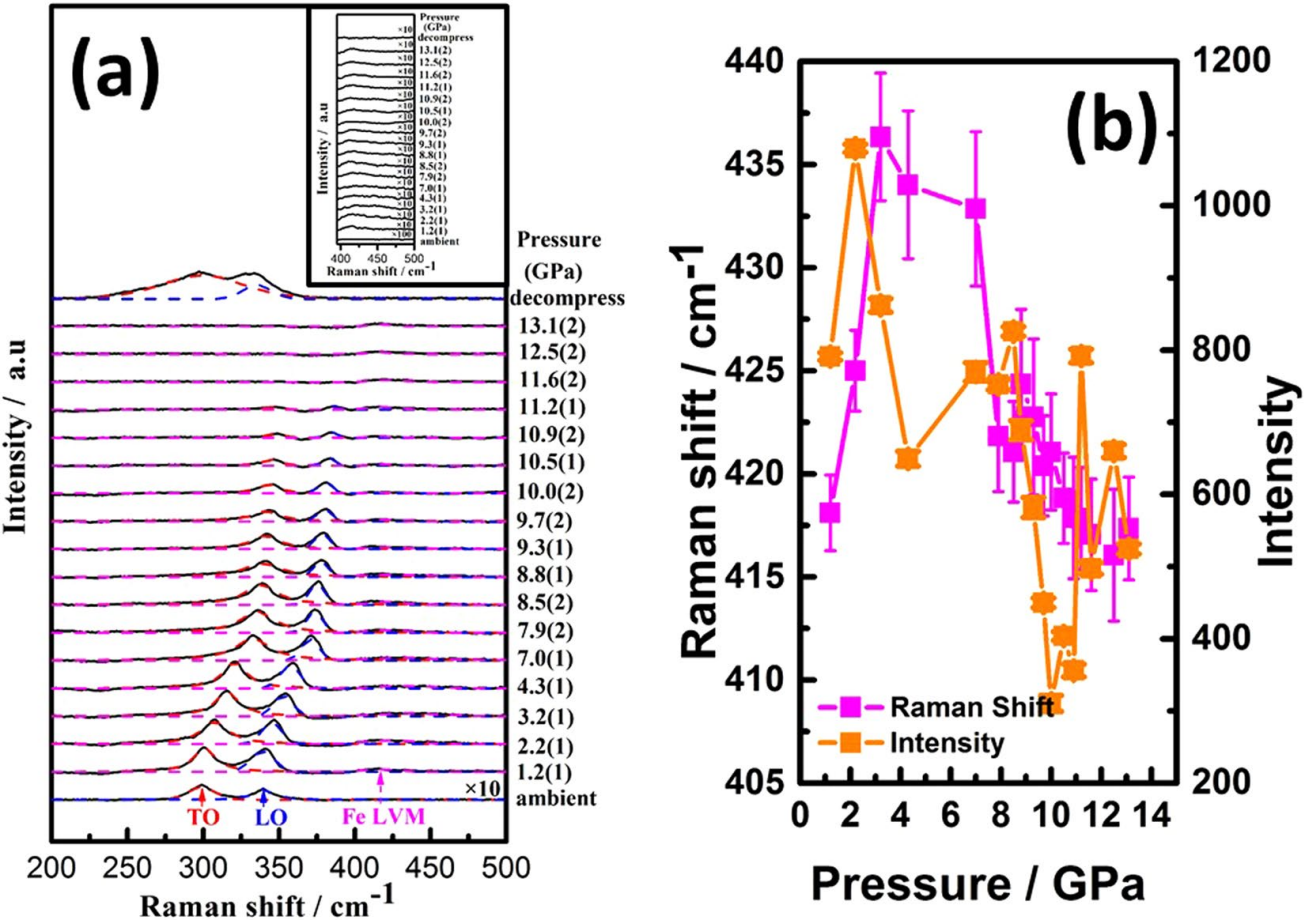
(**a**) Pressure dependence of phonon frequencies of bulk InP:Fe. (**b**) The frequency and intensity evolutions as a function of pressure for the Fe LVM mode.

**Figure 5:**
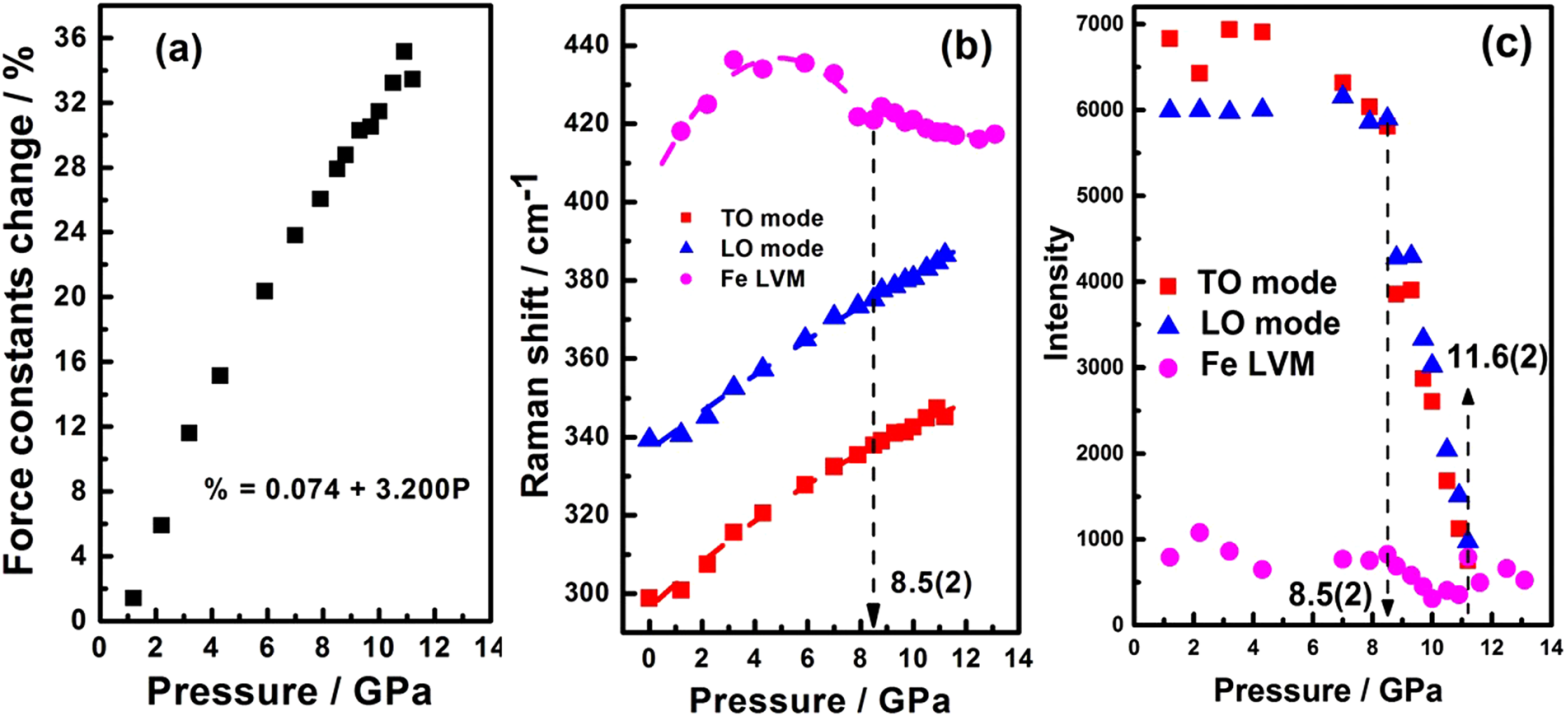
(**a**) The relationship of the percentage change in force constant (%) versus pressure (*P*) of TO phonon mode. (**b**) Mode frequencies of InP:Fe as functions of the pressure. (**c**) Intensity of the Raman modes of $F\bar{4}3m$ space group as a function of the applied pressure.

The doping of Fe in InP is expected to alter the local crystal symmetry and, thus, the behaviours of phonon mode, as well. Room-temperature Raman spectra for InP:Fe under various pressures are shown in Fig. 4(a). At ambient conditions, the positions of the zone center transverse optical (TO) and longitudinal optical (LO) phonon modes for InP:Fe are observed at 298.8 and 339.4 cm^−1^, which are slightly lower than that for pure InP, namely 304 and 345 cm^−1^ for TO and LO, respectively^31,32^. The Raman spectra at ambient conditions show no trace of BCC iron clusters possibly existing in the present InP:Fe, presumably due to its scarce amount as well as nanoscale fluctuating short-range nature. In fact, the BCC (α-Fe) phase has no first-order Raman spectrum because all the atoms sit on inversion centers. Newman^33^ indicated that, within the linear chain model, TO optical frequency at zero wave-vector $(\overrightarrow{k}=0)$ is also the maximum allowable frequency. Larkin^6^ reported that the vibrational frequency of the TO mode at $\overrightarrow{k}=0$ was: ${\omega }_{TO}=1303\sqrt{k(1/{M}_{In}+1/{M}_{P})}$, where *k* is force constant, which is a function of the energy of the In(Fe)-P atomic bonding. Based on these arguments, we obtain a force constant of 1.28 millidynes/Angstrom (md/A) at ambient pressure, which is significantly smaller than 3–6 md/A of single-bond molecular vibrations. At 1.2(1) GPa, the TO and LO phonon modes for InP:Fe are observed at 300.9 and 340.6 cm^−1^, respectively. It is noted that one additional relatively weak structure locating at 418.1 cm^−1^ can be identified through the de-convolution process, which has been labelled as Fe LVM^6,7^. The frequency and intensity evolutions as a function of pressure for the Fe LVM mode are shown in Fig. 4(b). It is evident that the pressure dependence of frequency and intensity are of the opposite trend, indicating the anharmonic nature of the Fe LVM mode. The inset of Fig. 4(a) shows the enlarged plot for Fe LVM. The appearance of LVM is likely due to a relaxation of the Raman selection rules associated with the iron-induced defects in the system^34^. Sun *et al*.^35^ from their Fourier transform infrared (FTIR) spectroscopy study on bulk n-type LEC InP had concluded that iron dopant was largely compensated by the fully hydrogenated indium vacancy, V_In_H_4_, which is related to the 2316 cm^−1^ LVM. When an iron atom is introduced into the InP lattice, the translational symmetry is broken and one or more new vibrational modes may appear. Göbel *et al*.^36^ reported that the Fe^3+^ defect had a LVM mode in the optical phonon band, which was strongly hybridized with host modes. Such strong hybridization leads the LVM and host modes to form states similar to degenerate states and, hence, makes it almost undetectable at ambient pressure. Nevertheless, at 1.2(1) GPa, the difference of the total enthalpy change (Δ°H) for ambient to 1.2(1) GPa, which may be estimated as follows^26^: Δ°H ≈ P(−ΔV) = 1.2 GPa × 0.224 Å^3^ × 6.02 × 10^23^/mole = 0.16 kJ·mole^−1^, may be adequate to result in the splitting of the degenerated LVM from the host modes. When an indium atom in the chain is replaced by an iron atom of smaller mass *M*_*Fe*_, the force constant will not change significantly, and the characteristic vibrational frequency becomes ${\omega }_{FeLVM}=1303\sqrt{k(1/\tau {M}_{P}+1/{M}_{Fe})}$, where *τ* is an empirical constant representing geometric details of the bonding interaction. Perkowitz^37^ reported that ${\omega }_{FeLVM} > {\omega }_{TO}$ and, since it is a LVM, the vibrational mode cannot propagate through InP:Fe crystal like ordinary phonons. As is evident from Fig. 4, when the applied pressure is further increased, no mode splitting on the LO and TO phonons can be found and both modes shift towards higher frequency side with gradually decreasing intensity. The pressure effects on the blue shift of LO and TO phonons are shown in Fig. 5(b). At 11.6(2) GPa, both TO and LO phonon modes become undistinguishable from the background. The disappearance of both TO and LO phonon modes has been attributed to the metallization effect of semiconductors under high pressure^38,39^. Moreover, such transition has also been considered as a manifestation of material being transformed from a direct band gap into an indirect one^13–15^.

At the pressure of 13.1(2) GPa (Fig. 4(a)), the Raman pattern shows no trace of hexagonal-close-packing (HCP) (ε-Fe) formed in the present InP:Fe. Takahashi *et al*.^40^ reported that α-Fe phase transforms to ε-Fe phase at 13 GPa by using XRD measurement under room temperature. Thus, if there were ordered BCC Fe existing in the sample, then two Raman bands are expected to appear around 210 and 250 cm^−1^ at 13 GPa, with the stronger band identified as the E_2g_ fundamental predicted by symmetry for the HCP lattice^41^. The absence of E_2g_ Raman phonon mode also provides a further evidence of lack of long-range-ordered Fe structure as revealed by the XANES and EXAFS results. After decompression, the positions of the TO and LO phonon modes for InP:Fe resumed at 299.3 and 335.6 cm^−1^. However, no Fe LVM was observed after decompression (Fig. 4(a)).

On the other hand, the pressure effect on the Fe LVM exhibited a rather interesting behavior. As shown in Fig. 4(a) and Fig. 5(b), the Fe LVM, originally indistinguishable at the ambient pressure, appears at the pressure of 1.2(1) GPa with a Raman shift of 420 cm^−1^. It then blue shifts with increasing pressure until reaches 440 cm^−1^ in the pressure range of 4–6 GPa (Fig. 5(b)) then drops back to 420 cm^−1^ when the applied pressure is further increased. To understand this interesting yet peculiar behavior, it is heuristic to compare how the force constant associated with the vibration modes varies with the applied pressure. Figure 5(a) shows the force constant changes of the TO phonon mode as a function of the applied pressure. The relationship of the percentage change in force constant (Δ*k*/*k*_0_*100%) versus pressure (*P*) of TO phonon mode can be obtained by the least-square linear fitting, which yields the expression: Δ*k*/*k*_0_*100% (*P*) = 0.074 + 3.200 *P*, where *k*_0_ is the force constant at ambient pressure. Ferraro^32^ indicated that the key factor in pressure-induced Raman frequency shifts was mainly due to the changes in force constant. Sherman *et al*.^42^ reported the percentage change in force constant was related to the percentage change in interatomic distance and found that all bond-stretching force constants were anharmonic. In our case of In(Fe)-P here, similar anharmonic chemical bond is found and the anharmonicity appeared to increase with increasing external pressure. The quadratic polynomial fitting, ${\omega }_{i}={\omega }_{0}+\omega ^{\prime} P+\omega ^{\prime\prime} {P}^{2}$, of mode frequencies as function of the pressure for InP:Fe shown in Fig. 5(b) yields:

1 $${\omega }_{TO{mode}}=296.48+6.099\,P\,-\,0.144\,{P}^{2},$$

2 $${\omega }_{LOmode}=336.76+5.001\,P\,-\,0.054\,{P}^{2},$$

3 $${\omega }_{FeLVM( < 8.5(2)GPa)}=403.87+13.384\,P-1.358\,{P}^{2},$$

4 $${\omega }_{FeLVM( > 8.5(2)GPa)}=508.72-14.813\,P+0.596\,{P}^{2},$$

where *ω*_*i*_ is the wave number in cm^−1^ and *P* is the pressure in GPa. Anharmonic properties of solids are usually represented in terms of the Grüneisen parameter *γ*. The Grüneisen parameter (*γ*_*i*_) for the *i*^th^ quasi-harmonic mode of frequency *ω*_*i*_ is defined as^43^:

5 $${\gamma }_{i}=-(\frac{d\,\mathrm{ln}\,{\omega }_{i}}{d\,\mathrm{ln}\,V\,})=\frac{1}{\beta }\frac{\partial \,\mathrm{ln}\,{\omega }_{i}}{\partial P}=(\frac{{B}_{0}}{{\omega }_{i}})(\frac{d{\omega }_{i}}{dP}),$$

where *B*_0_ is the zero-pressure isothermal bulk modulus for InP and is determined to be 72.5 GPa for the ZB structure by Trommer *et al*.^44^. The *β* parameter is the isothermal volume compressibility and *V* is the molar volume in cm^3^ · mole^−1^. The Grüneisen parameters for the TO, LO, Fe LVM (<8.5(2) GPa), and Fe LVM (>8.5(2) GPa) of InP:Fe are 1.49, 1.08, 2.40, and −2.11, respectively. The results demonstrate that the Raman shift of both the TO and LO modes varies pretty much quadratically with pressure, whereas the shift of the Fe LVM changes from a negative curvature to a positive curvature around the pressure (∼8.5(2) GPa) at which the ZB to RS structure transition occurs, suggesting that the peculiar pressure dependent behavior of Fe LVM seen in Fig. 5(b) is intimately correlated with the anharmonicity originated from Fe-doping, which ultimately affects pressure-induced phase transition of InP:Fe. Another interesting feature to be noted is, as shown in Fig. 5(c), how the intensity of all phonon modes varies with the applied pressure. The results evidently demonstrate that below 8.5(2) GPa the intensity of the LO and TO modes of ZB-structured InP:Fe decreases gradually and exhibits a discontinuous drop at 8.5(2) GPa. This pressure, 8.5(2) GPa, seen in Raman measurements apparently connects consistently with the onset transition pressure (8.2(2) GPa) of ZB to RS structure revealed in ADXRD measurements. On the other hand, although the intensity of the Fe LVM is comparatively low, it exhibits a rather irregular variation with the pressure, which is not clear at present. Nevertheless, it is noted that even beyond 11.6(2) GPa the Fe LVM is still visible, even though at this pressure the intensity both TO and LO modes have become diminishingly small due to the metallization effect (see below).

Briefly, by combining the results obtained from ADXRD and Raman measurements, a more detailed physical description on the pressure-induced phase transition in bulk InP:Fe over the pressure range of 8.2(2)–14.6(2) GPa can be given as follows. Beginning from 8.2(2) GPa at which the first appearance of (200) reflection of the RS phase is observed in ADXRD spectrum, indicating that it is the pressure of the onset of ZB to RS structural phase transition. From 8.2(2) to 10.4(1) GPa, the phase transition prevails continuously but rapidly, featuring the appearance of majority of site-ordered diffraction pattern of RS phase, the equal weight fraction value of ZB and RS phases and a discontinuous drop in the intensity of LO and TO phonon modes of the ZB phase. The transformation from ZB to RS structure is completed at 16.0(2) GPa, accompanied by a strong effect of metallization signified by the disappearance of TO and LO Raman phonon modes at 11.6(2) GPa. Moreover, the *ω*(*P*) for various characteristic modes shown in Fig. 5(b), namely the TO, LO, and the Fe LVM of InP:Fe indicate that below 8.5(2) GPa, all three Raman bands show nonlinear but smooth *ω*(*P*) characteristic. Beyond 8.5(2) GPa, changes in curvature and slope exist in *ω*(*P*) suggest that the interatomic distance of the Fe-P bond in the 4-fold coordinated tetrahedral sites in ZB structure is different from that in the 6-fold coordinated octahedral sites in RS structure. Thus, one may attribute the difference in the anharmonicity to the emergence of ZB to RS structural transformation in the entire InP:Fe specimen, which is consistent with the results obtained by ADXRD measurements.

Next we note that, unlike the pressure dependence of both TO and LO phonon modes, beyond 11.6(2) GPa, the Fe LVM is still visible up to the highest pressure (∼13 GPa) achieved in our Raman experiments. To understand this seemingly peculiar behavior, it is heuristic to compare the behaviors of Raman shifts beyond the metallization pressure observed in ZnSe:Fe DMS with similar crystal structure^36^, wherein the existence of Fe doping was found to result in a reduction in the semiconductor-metal phase transition pressure, albeit both LO and Fe LVM were disappeared upon the metallization pressure. In the present case, we may assume similarly that the permeability of the metallized InP:Fe is about the same as that of the InP under vacuum. The resistivity of InP at pressures above 11.6(2) GPa is reported to be smaller than 20–65 μΩ-cm^45^, giving rise to a calculated skin depth at pressures above 11.6(2) GPa of only about several tens of Å. Given the wavelength of 514.5 μm for the excitation laser beam, it is quite interesting to ask what makes the number of scattered photons, especially for the Fe LVM, become large enough to be detected for such a thin penetration depth. We believed that the anharmonic nature alone may not be adequate to comprehend the entire physical picture prevailing here and further investigations are certainly needed to address this interesting observation. Nevertheless, it is also noted that when the pressure is released back to the ambient pressure tremendous amount of strain remained in the sample, albeit the bulk InP:Fe has essentially reverted to the ZB from the metastable RS structure, as is evident from the drastic broadening of the corresponding Raman peaks displayed in Fig. 4. This suggests that the pressure effects on the Fe^3+^-induced defects might have been relevant to the observed pressure dependent behaviors of the Fe LVM.

### Summary

In summary, *in-situ* high pressure ADXRD and Raman scattering measurements have been systematically performed up to around 19.0(2) and 13.1(2) GPa, respectively, to investigate the pressure-induced phase transitions on bulk InP:Fe. The XANES and EXAFS measurements under ambient pressure showed that the oxidation state of iron is primarily Fe^3+^(3*d*^5^) and its local geometry (FeP_4_) is T_d_ symmetry with 2.28(1) Å of Fe-P bond lengths. The ADXRD measurements reveal that the onset pressure for the ZB-to-RS structural phase transition in the present InP:Fe system is ∼8.2(2) GPa, which is significantly smaller than the magnitude of over 10 GPa reported previously, indicating the prominent role played by Fe doping. From 8.2(2) to 10.4(1) GPa, the phase transition prevails continuously but rapidly, featuring the appearance of majority of site-ordered diffraction pattern of RS phase, the equal weight fraction value of ZB and RS phases and a discontinuous drop in the intensity of LO and TO phonon modes of the ZB phase. Although based on XRD data the structural transition is complete above 16.0(2) GPa, the Raman spectra of the TO and LO modes are significantly suppressed at pressure beyond 11.6(2) GPa. The fact that the compression rate along the ***a***-axis of ZB structure is higher than RS structure indicates that the ZB structure is more susceptible to compression than the RS structure. Increase of the external pressure is also found to increase the anharmonicity of In(Fe)-P bonds. The results also suggested that, at the ZB to RS structure transition pressure, the occupation of Fe^3+^ switches from tetrahedral to octahedral coordination, which gives the corresponding crystal field splitting energy parameters of Δ_*t*_ = 0.49 and Δ_*o*_ = 1.10 kJ·mole^−1^, respectively. The pressure-dependence of Fe LVM is believed to intimately correlate to the Fe-P bonding anharmonicity. The fact that such mode is detectable even when the estimated skin depth is far shorter than the wavelength of the excitation laser suggests that tremendous defects introduced during the pressurizing process might have been relevant in photon scattering. Nevertheless, a more comprehensive theoretical study is certainly needed to fully delineate this issue.

## Methods

### Determination of iron in indium phosphide wafer by inductively coupled plasma optical emission spectrometer (ICP-OES)

Concentration of iron in indium phosphide wafer was determined by inductively coupled plasma optical emission spectroscopy (ICP-OES, Thermo Fisher Scientific iCap 6500) and the matrix effect was corrected by standard sample addition as Kozono *et al*. works^46^. In this kind of analysis, the results of the same standard sample were 9 times better than 3% (2σ). Briefly, 0.1 gram of the sample was dissolved in 10 ml of concentrated hydrochloric acid solution, which was placed on a hot plate and covered by a lid. After digestion at 100 °C for ~50 hrs, the lid was removed and the solution was evaporated to dryness. The dried sample was then dissoved again in 0.3 N nitric acid and analyzed by the ICP-OES. All the sample pretreatment processes were carried out in a class 1000 clean room. The nitric acid and hydrochloric acid used were prepared by the double sub-boiling point distillation with purified reagent. The water used for preparing acid solutions was the Milli-Q ultra-pure water. The standard sample of iron was obtained from the High-Purity Standard company (North Charleston, South Carolina, USA). To determine the detection limit for iron element, we followed the method developed by Kozono *et al*.^46^. Namely, a standard with 50-times of the expected value and a blank were prepared. The instrument was allowed to stabilize and then 9 measurements of each solution were taken with 15 seconds of integration time. The detection limits were calculated using the raw intensity data from the standard and the blank. The detection limit for iron was 0.005 part per million (ppm)^46^. Although it is more than 20 times higher than the 0.25 part per billion (ppb) detection limit claimed in the specifications of the facility using standard sample introduction components consisting of a concentric nebulizer and cyclonic spray chamber^47^, but is more than adequate to resolve 1.02 ppm concentration of the present samples. The concentration of iron ([Fe]) in the 0.1 gram of sample was determined to be ~0.39 ppm. The atomic mass unit for iron (Fe_m_), indium (In_m_) and phosphorus (P_m_) are 55.847, 114.842 and 30.974, respectively. Using the formula

$$\frac{{{\rm{Fe}}}_{{\rm{m}}}\cdot {\rm{x}}}{{{\rm{Fe}}}_{{\rm{m}}}\cdot {\rm{x}}+{{\rm{In}}}_{{\rm{m}}}\cdot (1-{\rm{x}})+{{\rm{P}}}_{{\rm{m}}}}=[{\rm{Fe}}],$$

the value of x in In_1−x_Fe_x_P is about 1.02 × 10^−6^.

### Sample preparation

The parent (100)-oriented InP and iron-doped n-type (100)-oriented InP (InP:Fe) single crystal prepared by liquid encapsulated Czochralski (LEC) growth method were purchased from MTI Corporation. Iron doping forms midgap acceptor levels and results in significant carrier compensation, leading the host InP to become highly resistive SI material. The single crystal sample of In_1−x_Fe_x_P (x = 1.02 × 10^−6^) with a resistivity of 1 × 10^7^ Ω-cm and a low mobility of 64 cm^2^/V·sec was used in this study. Debney *et al*.^48^ pointed out that, since the production of iron-doped InP was frequently specified nominally by the amount of Fe added to a melt of undoped InP before the LEC process, the distribution of iron from the melt into the ingot was usually not well defined. Consequently, it is necessary to obtain direct chemical determination of the iron concentration on the actual n-type crystals used for the purpose of comparing the available electrical data. Mizuno *et al*.^49^ indicated that a relatively large amount of iron (∼0.15 wt%) would have been necessary in order to obtain a high resistivity of 10^7^ Ω-cm. Thus, the low carrier concentration (3–5 × 10^16^ cm^−3^) and the associated low mobility of InP:Fe might have been a consequence of the high iron doping. In the present study, the single crystal was ground into powder grains in a zirconium oxide ball mill with acetone for 2 h. After grinding, the powder was kept at room temperature for two weeks to release the possible residual stress resulting from the grinding process.

### X-ray absorption experiments and data analysis

To analyze the oxidation state of Fe in the semi-insulating InP:Fe, the X-ray absorption experiments were carried out at BL07A and BL17C beamlines at the National Synchrotron Radiation Research Center (NSRRC) in Hsinchu, Taiwan. The X-ray beam was monochromatized by a Si(111) double crystal monochromator with an energy resolution of ΔE/E ≈ 2 × 10^−4^. The Fe K-edge absorption spectra were taken in the fluorescence mode by a Lytle detector in the energy range 6912–7970 eV. The exact energy was calibrated with a simultaneous absorption measurement on the iron metal foil and used its first inflection point at 7112.0 eV for energy calibration. The EXAFS data analysis procedure is mainly based on the previous report published by Li *et al*.^50^. The AUTOBK program is used to do background subtraction and normalization to obtain EXAFS *χ*(*k*) function^51^. The *χ*(*k*) in the region 3.4 ≤ *k* ≤ 9.8 Å^−1^ is further weighted by *k*^2^ and then Fourier-transformed into the *R*-space as FT[*k*^2^χ(*k*)] to separate the backscattering contributions from different neighboring atoms. The EXAFS data analysis is done according to

6 $$\chi (k)={S}_{0}^{2}\sum _{j}\frac{{N}_{j}(k){F}_{j}(k)}{k{R}_{j}^{2}}sin[2k{R}_{j}(k)]{e}^{-\frac{2{R}_{j}}{\lambda }}{e}^{-2{k}^{2}{\sigma }_{j}^{2}},$$

based on the plane wave single scattering^52^, where *F*_*j*_(*k*) is the backscattering amplitude from each of the *N*_*j*_ atoms in the shell at distance *R*_j_ (relative to the absorbing atom), exp(−2*k*^2^σ_j_^2^) is the Debye-Waller factor with the mean-squared displacement *σ*_*j*_^2^, *S*_0_ is the amplitude reduction factor, *δ*_*j*_(*k*) is the total phase shift, and *λ*(*k*) is the photoelectron mean free path. With *S*_0_ fixed at 1.0 and the values of *F*_*j*_(*k*), *δ*_*j*_(*k*), and *λ*(*k*) calculated using a curved-wave *ab initio* procedure in the FEFF8 code^53^, we fitted FT[*k*^2^χ(*k*)] in the range 1.78 ≤ *R* ≤ 4.97 Å. For each phase, the fitting parameters are Δ*E*_0_, *R*_*j*_, *σ*_*j*_^2^, *N*_j_ and phase ratio *t*, using a nonlinear least-square fitting algorithm implemented by FEFFIT program^53^. In order to reduce the fitting variables, Δ*E*_0_ was confined to be the same for all scattering paths. Moreover, the coordination number (*N*_j_) were initially set to be a given value according to the standard crystallography structure of corresponding crystals, and then vary manually based on the best fitting restuls. However, the phase ratio *t* is always varied as a global parameter during the fitting process. Data fitting quality was evaluated with the goodness-of-fit factor defined as.

7 $${{\rm{R}}}_{{\rm{fit}}}=\sum _{i=1}^{n}\{{[{\rm{Re}}({{\rm{f}}}_{{\rm{i}}})]}^{2}+{[{\rm{Im}}({{\rm{f}}}_{{\rm{i}}})]}^{2}\}/\sum _{i=1}^{n}\{{[{\rm{Re}}({\tilde{{\rm{\chi }}}}_{{\rm{data}}{\rm{i}}})]}^{2}+{[{\rm{Im}}({\tilde{{\rm{\chi }}}}_{{\rm{data}}{\rm{i}}})]}^{2}\},$$

where $\tilde{\chi }={k}^{2}\chi$ and *n* is the number of evaluations of *f*_*i*_, with

8 $${f}_{i}={\tilde{\chi }}_{{\rm{data}}i}-{\tilde{\chi }}_{{\rm{model}}i}$$

(and hence *R*_*fit*_) minimized in the nonlinear least-square fitting algorithm (1, 2).

### Determination of the lattice parameters of the parent InP

Synchrotron powder X-ray diffraction data were collected at TPS 09 A (Taiwan Photon Source) of the National Synchrotron Radiation Research Center. The 15 keV X-ray source is delivered from an in-vacuum undulator (IU22) and the powder diffraction patterns were recorded by a position-sensitive detector, MYTHEN 24 K, covering a 2θ range of 120°. InP powder sample was loaded into a 0.3 mm capillary for uniform absorption and faster rotation during data collection. Due to the small gaps between detector modules, the two data sets were collected 2° apart with 60 seconds exposure time and the data were merged and gridded to give a continuous data set. Figure S2 shows the representative *in situ* ADXRD spectrum of bulk InP purchased from MTI Corporation. The unit-cell parameters and the interatomic bond length (bonding distance) in In-P at ambient pressure determined from Figure S2 are *a* = 5.8682(1) Å, V/Z = 50.518(3) Å^3^ (Z = 4) and 2.541(1) Å, respectively.

### Angle-dispersive X-ray diffraction under high pressure

The ground powder of the InP:Fe sample was loaded into a symmetric diamond anvil cell (DAC) as previously reported^21^. Pressure was measured using the ruby fluorescence technique^54^. The ADXRD measurements were performed using the beamline BL01C2 at the National Synchrotron Radiation Research Center (NSRRC), Taiwan, and beamline BL12B1 at SPring8, Japan. The wavelengths were 0.6199 Å (20 KeV) and 0.6191 Å (20.0273 KeV) for BL01C2 and BL12B1, respectively. Final values of the lattice parameters were obtained using the Rietveld refinement, which refined user-selected parameters by minimizing the difference between the experimental pattern and shape based on the composite crystal structure and instrumental parameters. Ten parameters, namely the scale factor, the 2^nd^ order polynomial background (3 parameters), ZB and RS lattice parameters (2 parameters), thermal parameters, occupancy factors, zero shift (specimen displacement), and profile shape parameters were used to refine information in this work. Thus, the DAC with diffraction angle 2θ up to 36° at 20.0273 keV can adequately match the required conditions of the Rietveld refinement. The weight fraction value (*y*) versus pressure (*x*) data is fitted to a Boltzmann function: y = A_2_ + (A_1_ − A_2_)/{1 + exp[(*x* − *x*_0_)/*dx*]}. Here, A_1_ and A_2_ are the left and right horizontal asymptote, respectively; *x*_0_ is the pressure at half-maximal weight fraction value (point of inflection); *dx* is a parameter describing the width (the change in *x* corresponding to the most significant change in *y* values) of the weight fraction value versus pressure relationship.

### Raman scattering measurements

Raman scattering measurements were performed with a confocal micro-Raman system (TRIAX 550). The 5145 Å line with a power of 0.6 W from the Spectra-Physics *Stabilite* 2017 6.0 W Argon ion laser was focused to about 2~4 μm diameter on the sample surface. The back-scattered signal was collected by a microscopic system and recorded with a JOBIN-YVON SPEX SPECTRUM ONE liquid nitrogen cooled charge-coupled diode (CCD) detector. All spectra were recorded with a Leitz UM 32 microscope objective and 3 accumulations at 1 second and 600 seconds integration time with ~50 mW power on the sample for each ruby fluorescence and Raman spectrum, respectively. The laser beam was focused to about 2∼4 μm on the sample surface and the excitation power density was estimated to be about 2.5 × 10^5^∼10^6^ W/cm^2^. Consequently, the high power density-induced local heating effect may lead to substantial thermal expansion and hence the observed frequency down-shift of the Raman scattering phonon modes. Wavenumbers are accurate to ±1 cm^−1^ as determined from plasma emission lines. The frequency of each Raman band reported in this study was obtained using Lorentzian curve fitting. The precision of the pressure determination with a corresponding resolution of DAC pressure of about 0.1–0.2 GPa was achieved by reading the peak position of the embedded ruby *R*_1_ and *R*_2_ fluorescence. The ADXRD and Raman data presented in this study were collected at room temperature and the pressure transmitting medium (PTM) used was the methanol-ethanol mixture with a 4:1 (in volume) ratio. The Jandel Scientific Peakfit computer program was used in a deconvolution process and determination of the band position, band intensity (i.e. band height), band area (i.e. integrated area) and band width (i.e. full width at half maximum, FWHM) of Raman spectra. Lorentz-Gauss cross product functions were used throughout and peakfitting was carried out until squared correlation coefficients with *r*^2^ greater than 0.995 were obtained.

### Determination of PTM in ADXRD and Raman measurements under high pressure

The pressure measurement involves recording the R_1_-line, which is a pure electronic quantum transition within the Cr^+3^ atom, luminescence spectrum of tiny crystals of ruby within the gasket hole. In particular, Piermarini *et al*.^55^ and Klotz *et al*.^56^ pointed out that the 4:1 methanol-ethanol mixture was the most commonly used PTM and had been investigated by a number of groups. Traditionally, the methanol-ethanol mixture with a 4:1 (in volume) ratio is known to be hydrostatic up to 10.5 GPa^56^. All of previous works conclusively showed that the glass transition of the PTM was at 10.5 GPa and the effect of deuteration occurred at 10.5 ± 0.5 GPa. However, our previous study showed that the R_1_-R_2_ splitting in the ruby fluorescence under the methanol-ethanol mixture with a 4:1 (in volume) ratio was maintained well up to 36.0 GPa^39^. Therefore, the non-hydrostatic components shouldn’t be a serious problem below 36.0 GPa under the 4: 1 methanol- ethanol liquid mixture. In the present study, for checking the hydrostaticity of PTM, such a splitting was well recorded up to 18.9 GPa under deionized water and 18.6 GPa under the 4: 1 methanol-ethanol liquid mixture, as shown in Figure S3(a) and (b), respectively. It is evident from Figure S3(a) that the R_1_-R_2_ splitting in the ruby fluorescence under deionized water is maintained well up to 18.9 GPa. Consequently, as mentioned above, the 4:1 methanol-ethanol mixture should be a suitable PTM for the present high-pressure study.

## Electronic supplementary material

Supplementary information
