# Supplementary material for: Pressure effect on impurity local vibrational mode and phase transitions in n-type iron-doped indium phosphide
Source: Sci Rep. 2018 Jan 19;8:1284. doi: 10.1038/s41598-018-19679-2 (PMC5775340; doi:10.1038/s41598-018-19679-2)

Pressure effect on impurity local vibrational mode and phase transitions in *n*-type iron-doped indium phosphide

Chih-Ming Lin[1*], I-Jui Hsu[2*], Sin-Cheng Lin[1], Yu-Chun Chuang[3], Wei-Ting Chen[2], Yen-Fa Liao[3] and Jenh-Yih Juang[4*]

[1]*Department of Physics, National Tsing Hua University, Hsinchu 30013, Taiwan*

[2]*Department of Molecular Science and Engineering, National Taipei University of Technology, Taipei 10608, Taiwan*

[3]*National Synchrotron Radiation Research Center, Hsinchu 30076, Taiwan*

[4]*Department of Electrophysics, National Chiao Tung University, Hsinchu 30050, Taiwan*

Captions of figures

Figure S1 The 1st derivative of Fe K-edge XANES of $In_{1-x}Fe_xP$ ($x = 1.02\times10^{-6}$) and some reference samples.

Figure S2 Representative ADXRD pattern of parent bulk InP at ambient pressure.

Figure S3 The ruby fluorescence patterns vs pressure in a pressure medium of (a) methanol-ethanol mixture with a 4:1 (in volume) ratio and (b) deionized water, respectively. The left-hand and right-hand side peaks of the plots present R_2 and R_1 peaks of the ruby fluorescence, respectively.

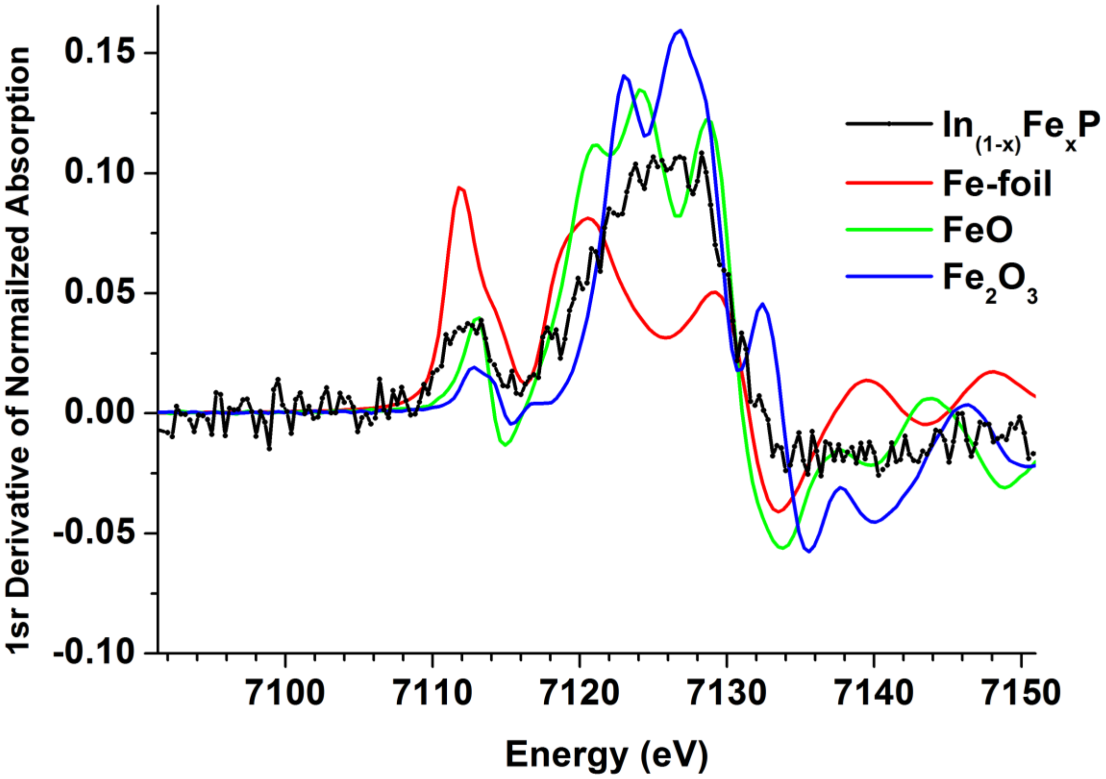

0.15
0.10
0.05
0.00
-0.05
-0.10
1sr Derivative of Normailzed Absorption
7100
7110
7120
7130
7140
7150
Energy (eV)
In(1-x)FexP
Fe-foil
FeO
Fe2O3

Fig. S2

C.M. Lin *et al.*

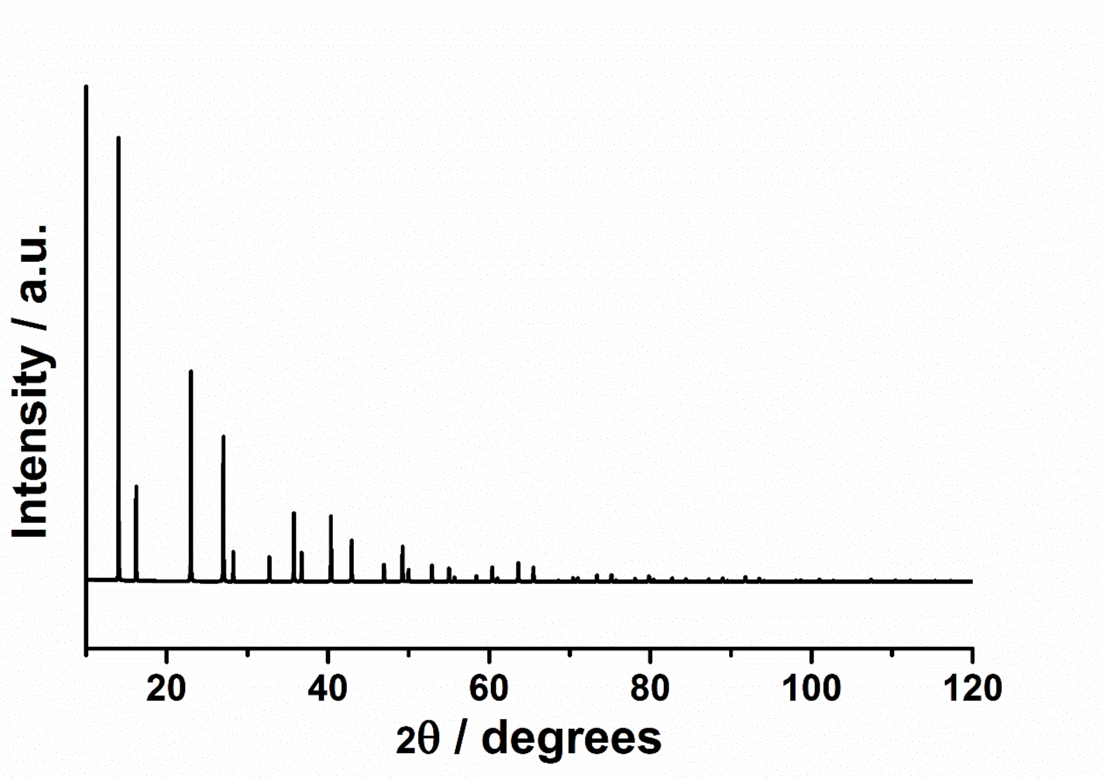

Fig. S2

C.M. Lin *et al.*

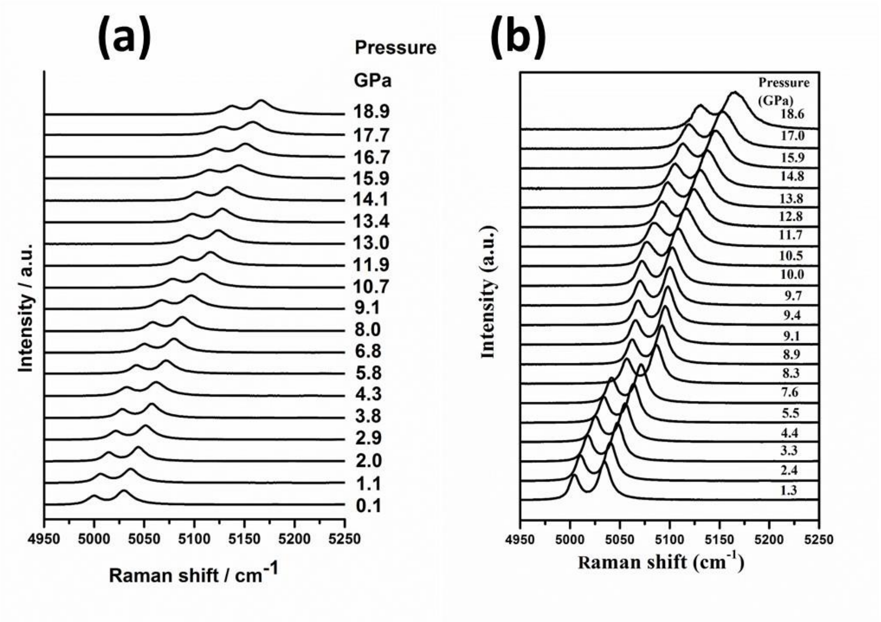

Supplement: Supplementary file 1 — Supplementary information [file 41598_2018_19679_MOESM1_ESM.pdf]
